# Supplementary material for: ProteinShader: illustrative rendering of macromolecules
Source: BMC Struct Biol. 2009 Mar 30;9:19. doi: 10.1186/1472-6807-9-19 (PMC2672931; doi:10.1186/1472-6807-9-19)
Supplement: Additional file 1 — ProteinShader program without source code. This compressed file contains the complete ProteinShader program including associated libraries, but no source code. A README.txt file gives an overview of the ProteinShader distribution, and the index.html file in the help subdirectory has directions on getting started with the program as well as a set of tutorials. [file 1472-6807-9-19-S1.zip › ProteinShader-beta-0_9_4-binary/help/api/org/proteinshader/structure/enums/class-use/BondEnum.html]

Uses of Class org.proteinshader.structure.enums.BondEnum (ProteinShader API)


|  |  |  |
| --- | --- | --- |
| **Overview** \| **Package** \| **Class** \| **Use** \| **Tree** \| **Deprecated** \| **Index** \| **Help** | |  |
| PREV   NEXT | **FRAMES**    **NO FRAMES**     **All Classes** |  |


---


## **Uses of Class org.proteinshader.structure.enums.BondEnum**

| Packages that use BondEnum | |
| --- | --- |
| **org.proteinshader.structure** | Holds the classes that store information from a Protein Data Bank file: Structure, Model, Chain, AminoAcid, Heterogen, Water, Atom, Bond, Helix, BetaStrand, Loop, *etc*. |
| **org.proteinshader.structure.enums** | Holds the enums needed by org.proteinshader.structure and its subpackages. |

Uses of BondEnum in org.proteinshader.structure

| Methods in org.proteinshader.structure that return BondEnum | |
| --- | --- |
| `BondEnum` | `Bond.getType()`             Returns the Bond's type as an enum (SINGLE, DOUBLE, PEPTIDE, *etc.*). |

| Methods in org.proteinshader.structure with parameters of type BondEnum | |
| --- | --- |
| `void` | `Atom.addNewBond(Atom dstAtom, BondEnum type, VisibilityEnum visibility)`             Adds a new Bond with this Atom as the source Atom and the Atom given as an argument as the destination Atom. |
| `void` | `Bond.setType(BondEnum type)`             Sets the Bond's type as an enum (SINGLE, DOUBLE, PEPTIDE, *etc.*). |

Constructors in org.proteinshader.structure with parameters of type BondEnum

`Bond(Atom srcAtom,
Atom dstAtom,
BondEnum type,
VisibilityEnum visibility)`
  
          Constructs a Bond for the source and destination Atoms given as
arguments.

Uses of BondEnum in org.proteinshader.structure.enums

| Methods in org.proteinshader.structure.enums that return BondEnum | |
| --- | --- |
| `static BondEnum` | `BondEnum.valueOf(String name)`             Returns the enum constant of this type with the specified name. |
| `static BondEnum[]` | `BondEnum.values()`             Returns an array containing the constants of this enum type, in the order they're declared. |

---


|  |  |  |
| --- | --- | --- |
| **Overview** \| **Package** \| **Class** \| **Use** \| **Tree** \| **Deprecated** \| **Index** \| **Help** | |  |
| PREV   NEXT | **FRAMES**    **NO FRAMES**     **All Classes** |  |


---

# *Copyright © 2007-2008*
